# Longitudinal evolution of non-motor symptoms in early Parkinson’s disease: a 3-year prospective cohort study

**DOI:** 10.1038/s41531-021-00207-5

**Published:** 2021-07-15

**Authors:** Ruwei Ou, Yanbing Hou, Qianqian Wei, Junyu Lin, Kuncheng Liu, Lingyu Zhang, Zheng Jiang, Bei Cao, Bi Zhao, Wei Song, Huifang Shang

**Affiliations:** Department of Neurology, Laboratory of Neurodegenerative Disorders, National Clinical Research Center for Geriatrics, West China Hospital, Sichuan University, Chengdu, Sichuan China

**Keywords:** Neurology, Parkinson's disease

## Abstract

The progression of global non-motor symptoms (NMS) in Chinese patients with Parkinson’s disease (PD) has not been explored. We aimed to examine the longitudinal evolution of overall NMS in a 3-year prospective Chinese cohort with early-stage PD. We included 224 patients with early PD who underwent annual evaluation of motor and non-motor symptoms. NMS was assessed using the non-motor symptoms scale (NMSS). We observed an increased number of NMS in the majority of the NMSS domains except mood/apathy and sexual dysfunctions. Significant deterioration was observed in the sleep/fatigue, perceptual problems/hallucinations, attention/memory, gastrointestinal, urinary, and miscellaneous domains during the follow-up (*P* < 0.05). Notably, the number and the score of sexual dysfunctions decreased with the progression of the disease. All NMSS domains showed a small effect size from baseline to 1-, 2-, and 3-year follow-ups (effect size < 0.5). The generalized estimating equations model indicated that the total number of NMS was significantly associated with age and the Unified Parkinson’s Disease Rating Scale (UPDRS) III score (*P* < 0.05). Multiple logistic regression indicated that a high number of NMS at baseline was associated with a 3-point, a 6-point, and a 9-point increase in the UPDRS III score from baseline to 1-year (odds ratio [OR] 1.074, *P* = 0.017), 2-year (OR 1.113, *P* = 0.001), and 3-year (OR 1.117, *P* < 0.001), respectively. Our study indicated that overall NMS evolution in early PD is mild and multidimensional; a high NMS burden in early PD predicts the faster motor progression of PD. Our study is helpful for understanding the longitudinal evolution of NMS in PD.

## Introduction

Non-motor symptoms (NMS) are frequently reported in patients with Parkinson’s disease (PD), which can occur either in the early stage or in the advanced stage of PD and may even precede the motor symptoms of the disease^1^. NMS are gaining increasing relevance in the management of PD, as they can greatly contribute to significant disability and reduced health-related quality of life^2,3^. In addition, NMS are considered as one of the frequent causes of hospitalization and institutionalization, which can increase the burden and the cost of care for patients with PD^4^.

A growing number of cross-sectional and case-control studies have explored the clinical characteristics of NMS in early PD, consistently demonstrating a high prevalence across the spectrum of NMS and a correlation with the degree of motor disability^5,6^. Our previous cross-sectional study^7^ found that age, disease duration, and motor disability contribute to the severity of NMS. In addition, a previous case-control study^8^ found that NMS are present throughout all stages of PD and their number increases with the severity of the disease and age.

Several studies have reported the progression of global NMS in patients with PD, but the results are inconsistent. Two studies^9,10^ predicted a significant increase in NMS overtime in early PD, while another study^11^ observed that the frequency of NMS tends to remain stable over time in early PD. Differences in genetic background, assessment tools, and study designs might have contributed to such discrepancies. To date, the early dynamic trajectory of overall NMS in Chinese patients with PD has not yet been reported. Further research involving the Asian population is warranted to gain a deeper understanding about the longitudinal evolution of NMS in patients with PD, as current evidence suggests that there are ethnic differences in the NMS burden.

In the present prospective cohort study, we recruited a group of patients with early PD who underwent a 3-year follow-up to determine the longitudinal evolution of NMS.

## Results

### Baseline and follow-up data

The demographic and clinical features of the patients with PD included in the study are listed in Supplementary Table 1. We included 224 PD patients at baseline (121 men and 103 women), and 195 patients completed the annual visits for 3 years. The mean age at baseline was 57.6 (standard deviations [SD] 11.1) years, with a mean disease duration of 1.5 (SD 0.9) years. The levodopa-equivalent daily dose (LEDD) was 129.3 (SD 175.5) mg/day at baseline and 468.2 (SD 255.8) mg/day after 3 years. One hundred and twenty-six (56.3%) patients were drug-naïve at baseline.

### Evolution of NMS

**Fig. 1:**
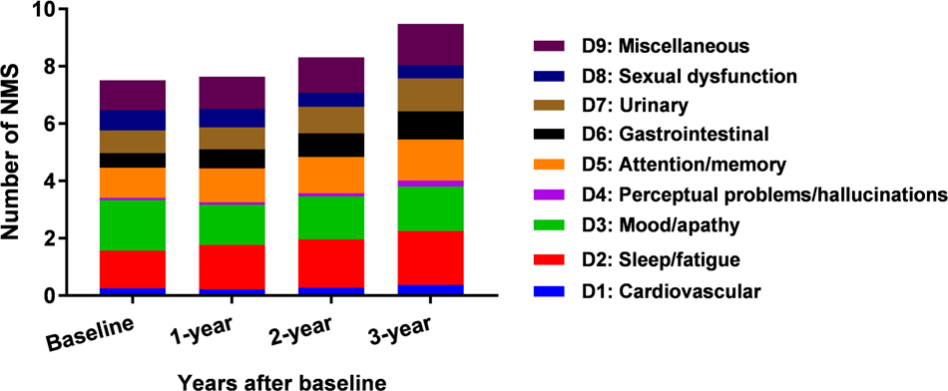
Number of NMS in patients with PD at each visit. The mean number of NMS in PD increased from 7.5 (SD 4.9) at baseline to 9.5 (SD 5.2) after 3 years. The composition of the number of NMS at each visit was presented in Fig. 1 as well. Increased number of NMS were observed in D1 (mean 0.3–0.7), D2 (mean 1.3–1.9), D4 (mean 0.1–0.2), D5 (mean 1.1–1.4), D6 (mean 0.5–1.0), D7 (mean 0.8–1.2), and D9 (mean 1.0–1.4) domains from baseline to 3-year. The number of NMS in the D8 domain was decreased from mean 0.7 at baseline to mean 0.5 after 3 years. Abbreviations: NMS non-motor symptoms, PD Parkinson’s disease.

The mean total number of NMS increased from 7.5 (SD 4.9) at baseline to 9.5 (SD 5.2) after 3 years (Fig. 1), with an increase in the NMSS score from 28.0 (SD 24.2) to 31.9 (SD 28.8) (Supplementary Table 1).

In the overall PD population, we observed that in most of the NMSS domains, including cardiovascular, sleep/fatigue, perceptual problems/hallucinations, attention/memory, gastrointestinal, urinary, and miscellaneous, the number of NMS increased across visits during the 3-year study period (Fig. 1). The number of sexual dysfunctions decreased from baseline to the 3-year follow-up (Supplementary Figure 1), while the number of mood/apathy was irregular (Fig. 1). The results from the analysis of the drug-naïve population at baseline were consistent.

**Fig. 2:**
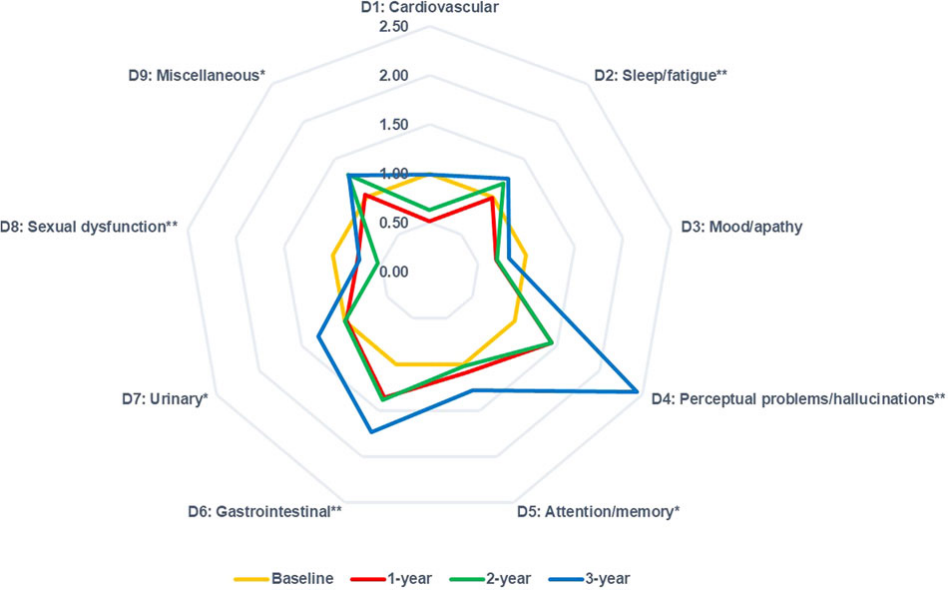
Comparison of NMSS domain scores at baseline, 1-, 2-, and 3-year. Illustrates: NMSS domain scores at baseline (golden), 1-year follow-up (red), 2-year follow-up (green), and 3-year follow-up (blue). In the radar chart, all values were normalized to baseline values (mean follow-up score/mean baseline score), and baseline values for all the NMSS domains were set to 1.0. Significantly increased scores were observed in D2, D4, D5, D6, D7, and D9 domains, while significantly decreased scores were observed in the D8 domain. Bigger areas compared to baseline represented more severe NMS at follow-up visits. Abbreviations: NMSS, Non-Motor Symptoms Scale; NMS, non-motor symptoms. *P < 0.05 and **P < 0.01 based on Friedman test.

In the overall population, significantly increased scores were observed in the sleep/fatigue, perceptual problems/hallucinations, attention/memory, gastrointestinal, urinary, and miscellaneous domains, while a significantly decreased score was observed in the sexual dysfunctions domain (Fig. 2). All domains showed a small effect size (ES) (< 0.50) from baseline to 1-, 2-, and 3-year follow-ups (Table 1). The results from the analysis of the drug-naïve population at baseline were consistent.

**Table 1:** Relative changes and effect sizes from baseline to 1-, 2-, and 3-year follow-up.

	Baseline to 1-year follow-up		Baseline to 2-year follow-up		Baseline to 3-year follow-up	
	RC (%)	ES	RC (%)	ES	RC (%)	ES
NMSS total score	−8.87	0.09	−2.94	0.03	12.09	−0.16
D1: Cardiovascular	−93.33	0.17	−58.18	0.13	−1.16	0.00
D2: Sleep/fatigue	−1.51	0.01	14.87	−0.14	19.46	−0.20
D3: Mood/apathy	-45.69	0.20	−43.35	0.19	−22.31	0.11
D4: Perceptual problems/hallucinations	30.43	−0.10	30.43	−0.10	58.97	−0.34
D5: Attention/memory	8.58	−0.07	2.12	−0.02	21.75	−0.20
D6: Gastrointestinal	26.36	−0.21	27.76	−0.22	42.42	−0.43
D7: Urinary	−2.13	0.01	−0.60	0.00	23.69	−0.19
D8: Sexual function	−34.55	0.15	−87.01	0.27	−37.34	0.15
D9: Miscellaneous	2.20	−0.02	22.63	−0.24	21.88	−0.23

All domains showed small effect size from baseline to 1-, 2-, and 3-year follow-up.

*RC* relative changes, *ES* effect size, *NMSS* non-motor symptoms scale.

Since the number and score of sexual dysfunctions in early PD decreased with disease progression, we further analyzed the differences in clinical features between patients with and without sexual dysfunctions at each visit to help understand this result. Patients with sexual dysfunctions had significantly higher Hamilton Depression Rating Scale (HAMD) and Hamilton Anxiety Rating Scale (HAMA) scores at baseline; a lower percentage of dopamine agonist use at 1-year; higher HAMD and HAMA scores at 2-year; and a higher percentage of the male sex, a higher proportion of levodopa use, as well as a higher Unified Parkinson’s Disease Rating Scale (UPDRS) III, Hoehn and Yahr (H&Y) stage, HAMD, and HAMA scores at 3-year than those without sexual dysfunctions (Supplementary Table 2). The Generalized estimating equations (GEE) model indicated that sexual dysfunction in PD was significantly associated with short disease duration, male sex, and high HAMD score (Supplementary Table 4). The adjustments for levodopa, dopamine agonist, and antidepressant use were presented in Supplementary Table 3.

### Factors associated with the number of NMS

The fitted regression lines indicated that the number of NMS increased with the UPDRS III score (Supplementary Figure 2). The GEE analyses indicated that the number of NMS was significantly associated with age (B 1.112, 95% confidence interval [CI] 1.044–1.184, *P* = 0.001) and UPDRS III score (B 1.124, 95% CI 1.053–1.201, *P* < 0.001) (adjusted model) (Table 2). In the sub-analysis of drug-naïve patients at baseline, the number of NMS was significantly associated with age (B 1.082, 95% CI 1.013–1.155, *P* = 0.019) and UPDRS III score (B 1.103, 95% CI 1.060–1.147, *P* < 0.001) (adjusted model).

**Table 2:** Factors associated with the number of NMS in patients with PD.

	Unadjusted model			Adjusted model		
	B	95%CI	*P*-value	B	95%CI	*P*-value
Female sex	0.153	0.037–0.628	0.009*	0.416	0.110–1.571	0.196
Age	1.145	1.074–1.221	<0.001*	1.112	1.044–1.184	0.001*
Disease duration	1.573	1.007–2.458	0.047*	1.078	0.684–1.698	0.746
Education	0.971	0.818–1.154	0.741	1.045	0.891–1.225	0.588
LEDD	1.005	1.002–1.008	0.001*	1.002	0.998–1.006	0.360
Use of levodopa	10.912	1.768–67.357	0.010*	2.024	0.213–19.242	0.539
Use of dopamine receptor	1.636	0.275–9.755	0.589	0.421	0.066–2.676	0.359
Use of antidepressant	3.087	0.214–44.556	0.408	2.682	0.235–30.662	0.427
UPDRS III	1.172	1.097–1.251	<0.001*	1.124	1.053–1.201	<0.001*

*Significant difference.

*NMS* non-motor symptoms, *PD* parkinson’s disease, *LEDD* levodopa equivalent daily dose, *UPDRS* unified parkinson’s disease rating scale.

### Number of NMS and motor progression

The forward binary logistic regression indicated that a higher number of NMS at baseline was significantly associated with a 3-point increase in the UPDRS III from baseline to 1-year (odds ratio [OR] 1.074, 95%CI 1.013–1.139, *P* = 0.017), a 6-point increase from baseline to 2-year (OR 1.113, 95%CI 1.048–1.182, *P* = 0.001), and a 9-point increase from baseline to 3-year (OR 1.117, 95%CI 1.048–1.191, *P* < 0.001) after adjusting for sex, age at enrollment, age of onset, disease duration, and UPDRS III score at baseline (Table 3).

**Table 3:** Association between baseline NMS number and motor progression in PD.

	Number of NMS at baseline	
	OR (95%CI)	*P*-value
UPDRS-III 3-point increase from baseline to 1-year	1.074 (1.013–1.139)	0.017*
UPDRS-III 6-point increase from baseline to 2-year	1.113 (1.048–1.182)	0.001*
UPDRS-III 9-point increase from baseline to 3-year	1.117 (1.048–1.191)	<0.001*

* Significant difference.

In the multivariate models, sex, age at enrollment, age of onset, disease duration, and UPDRS III score at baseline were adjusted.

*PD* parkinson’s disease, *NMS* non-motor symptom, *UPDRS* unified Parkinson’s disease rating scale.

## Discussion

Our study explored the longitudinal evolution of NMS in a large sample of Chinese patients with early PD. In the 3-year prospective longitudinal study, we observed an increased number and severity of NMS in the majority of the NMSS domains overtime in early PD. However, the longitudinal changes were small (ES < 0.50). Notably, the number and score of sexual dysfunctions decreased. The high burden of NMS at baseline was associated with faster motor progression of PD. Our results demonstrated that the progression of NMS in early PD is mild and multidimensional, and the findings have potential implications for understanding the longitudinal evolution of NMS in PD.

A major novel aspect of this study is that we used a comprehensive tool (the NMSS) to explore the early progression of overall NMS in Chinese patients with PD for the first time. An Italian study^11^ used the non-motor symptoms questionnaire to assess the 2-year change in NMS before and after starting therapy in newly diagnosed PD patients and found that the frequency of NMS tended to remain stable during the early phase of the disease. This observation was inconsistent with our current finding, which suggested that the number and score in most of the NMS increased with disease progression. Differences in the genetic backgrounds and treatment strategies might have contributed to such discrepancies. In our previous cross-sectional study^12^, we found that NMS was prevalent in PD patients irrespective of the disease duration, and the severity of NMS increased with the prolonged disease. In addition, an American study^10^ observed a significant increase in NMS over 2-year in early untreated PD when compared with healthy controls. Another European multicenter study^13^ found that the prevalence of nearly all NMS increased significantly during the early stage of PD despite patients being on the “best medical treatment”, which also supports NMS progress in early PD. Accumulated evidence indicates that early management of NMS in PD is necessary since the severity of NMS has been reported to exhibit a high association with reduced quality of life^2,3^. However, not all NMS domains showed an increase over time in our cohort, indicating that the underlying biological underpinnings of these NMS symptoms are different. Future evaluation of our cohort after a longer follow-up will further help in determining whether NMS progression continues beyond the first 3-years after the diagnosis.

Sexual dysfunction is recognized as a later manifestation of autonomic failure in PD which may help to differentiate it from other parkinsonian syndromes such as Multiple system atrophy. Interestingly, in the early stage of PD, we found that the number and score of sexual dysfunction tended to become decrease with the progression of the disease. Some explanations for such novel findings can be considered. Depression has been proven to be a probable contributor to decreased sexual desire in PD^14^. In the present study, we observed that higher HAMD scores were associated with sexual dysfunction (Supplementary Table 4). Thus, we suspect that the change in the HAMD score observed in our cohort (Supplementary Table 1) might have had a potential effect on the improvement of sexual dysfunction. Moreover, many patients are likely to regard sexual dysfunction as a normal issue associated with increased age, which might also contribute to the decreased number of sexual dysfunction reported in our cohort. Although no link was observed between the change in drug use and sexual dysfunction (Supplementary Tables 3 and 4), we still speculate that the start of pharmacological therapy after enrollment might have affected the decreased report in the number of sexual dysfunctions. However, we did not investigate the social and cultural factors affecting the development of sexual dysfunctions in the present study. Factors such as personality and religious faith have been reported to be associated with sexual dysfunction^15^. Hence, further prospective studies with longer follow-up using validated scales to assess sexual function considering more comprehensive factors need to be performed while addressing sexual dysfunction in all disease stages of PD.

In the present study, the overall number of NMS in patients with PD increased with motor severity at each visit. Our study delivered consistent results with a previous study^5^ that used a 12-domain semi-structured interview. More importantly, we found that the high burden of NMS at baseline predicted faster motor progression of PD in our cohort, which has implications for predicting motor progression in early PD. As described by Braak and colleagues, the increased number of NMS in PD represents an extensive spread of Lewy bodies in PD^16^, suggesting that patients with a higher burden of NMS may exhibit broader damage of the dopaminergic cell.

Dopaminergic and non-dopaminergic neurotransmitter deficits have been reported to be involved in the pathogenesis of NMS in PD^16^. The association between motor severity and NMS burden in our cohort suggests a common pathophysiological mechanism. We inferred that the degree of dopamine deficiency plays a role in this mechanism. The partial improvement in some NMS such as depression, bladder function, pains, and sleep disorders by dopaminergic treatment also suggests some degree of non-motor impairment are driven by dopaminergic denervation^4,17^. An 18F-dopa positron emission tomography study^18^ provided evidence of dopaminergic deficits in the hypothalamus in PD contributing to the development of NMS such as sleep, endocrine, and autonomic disorders in PD.

However, the lack of association between NMS and dopaminergic drugs observed in our cohort and the clinical observation that NMS cannot be completely relieved by dopamine replacement therapy in PD indicate that dopaminergic deficit is not a unique pathophysiological mechanism contributing to NMS. A 4-year longitudinal study^13^ on the baseline prevalence and longitudinal evolution of NMS found that the progression of most of the NMS lacks associations with LEDD and motor disability, as measured by the UPDRS III. Lack of association with the motor disability in advanced PD cohorts as well as in de novo PD population is also reported by others^19–21^. This evidence supports the notion that deficits in non-dopaminergic neurotransmitters such as serotonin and noradrenaline may also contribute to the various non-motor signs and symptoms of PD.

Age was a risk factor for an increased burden of NMS in our cohort. The association between age and NMS in PD has also been observed in our previous cross-sectional study^7^ and in another case-control study^22^. Additionally, a cross-sectional study^23^ also revealed that age was associated with constipation, orthostatic hypotension, urinary incontinence, and erectile dysfunction in male patients with PD. The association between age and NMS burden was probably due to the contribution of older age to the faster progression of motor disability^24^, less response to levodopa treatment^25^, and more severe cognitive impairment^26^ in patients with PD. However, disease duration did not contribute to an increased number of NMS in our cohort. We speculate that the effect of disease duration on the NMS burden in the early stage of PD is relatively weak and it can be covered by increased age and motor progression since the overall NMS evolution is relatively mild along with prolonged disease duration in our cohort.

Following are the limitations of the study: (1) The study did not include a group of healthy individuals against whom we could compare the progression of NMS in patients with PD. (2) The relatively short observation period of disease progression in some patients was insufficient to determine the long-term evolution of NMS in PD. (3) We did not consider the influence of non-motor fluctuations in the present study.

In conclusion, our study showed that the number and severity of NMS increased in patients with early PD. However, we observed that sexual dysfunctions improved with the progression of the disease. Our study highlighted that the progression of NMS is mild and multidimensional in the early phase of PD, and a high burden of NMS in the early phase accelerates the motor progression of PD. Our results have implications for future drug studies and clinical trials targeting NMS.

## Methods

### Study design and population

The study was approved by the Ethics Committee of Sichuan University West China Hospital and written informed consent was obtained from all participants.

In this ongoing prospective longitudinal cohort study performed at the Department of Neurology, Sichuan University West China Hospital, we aimed to investigate the prognosis and progression of PD in southwest China. This project was initiated in February 2014 and aimed to recruit patients with early PD (*n* = 302). PD was diagnosed based on the United Kingdom PD Society Brain Bank clinical diagnostic criteria^27^. All subjects recruited at baseline had a disease duration of < 3 years. The exclusion criteria were as follows: (1) patients with cognitive impairment, as assessed by the Beijing version of the Montreal Cognitive Assessment (MOCA) tool with a cut-off score < 22^28^, (2) patients with motor fluctuations and dyskinesia, as scored ≥ 1 on the UPDRS part IV, and (3) patients with H&Y stage ≥ 3.

All patients underwent standardized examinations and regular assessments by trained neurologists annually at our movement disorder center. The examinations and assessments were performed at baseline, as well as 1-, 2-, and 3-year follow-ups. Among the 302 patients recruited initially, 78 were excluded owing to lack of assessment using the NMSS at baseline. Thus, 224 patients were eligible for inclusion in the study.

### Evaluation protocol

Baseline demographic and clinical data including sex, age, age of disease onset, disease duration, and educational level were recorded. The therapeutic regimen was recorded during each visit. The total LEDD was calculated based on a previous report^29^.

All recruited patients underwent a repeated series of neurological evaluations at baseline and during follow-up. The severity of motor symptoms was evaluated using the UPDRS part III^30^ and H&Y stage^31^. The HAMD, which contains 24 items^32^, was used to evaluate depression, and the HAMA^33^ was used to evaluate anxiety. Executive function was evaluated using the Frontal Assessment Battery (FAB)^34^. The Beijing version of the MOCA screening tool was used to assess the global cognitive function^35^.

NMS were evaluated annually using the Chinese version of the NMSS^36^, a validated scale to assess NMS in PD. The NMSS contains nine domains: cardiovascular, sleep/fatigue, mood/apathy, perceptual problems/hallucinations, attention/memory, gastrointestinal, urinary, sexual dysfunction, and miscellaneous. The severity of each item from the NMSS was calculated using the product of both frequency (1–4) and severity (0–3). A score ≥ 1 in any of the NMSS items indicates that the patient had the respective NMS. The total number of NMS was calculated as the sum of the number of NMS reported by the patients at each visit.

### Outcome definition

A change of 2.5–5.2 points in the UPDRS III score was reported as a clinically significant difference^37^. Therefore, we defined fast motor progression as a 3-point increase in the UPDRS III per year.

### Statistical analyses

All statistical analyses were performed using the IBM SPSS Statistics (version 22.0). Statistical tests were two-tailed, and *P*-values < 0.05 were considered statistically significant. Continuous variables were reported as means and SD, and categorical variables were presented as counts (percentages) or median (quartiles). Student’s *t*-test, Χ^2^-test, Fisher’s exact test, and Mann–Whitney U test were used for intergroup comparisons of clinical variables.

The GEE model with multiple linear regression analysis were used to determine the factors associated with the number of NMS. The models were used to explore the correlation between repeated measurements obtained from the same patients, which included patients with consecutive evaluations over the follow-up period. An exchangeable working correlation structure was selected. The number of NMS was set as the dependent variable (continuous variable) in the model. The independent variables included the following repeated measures: sex, age, disease duration, educational level, LEDD, use of levodopa, use of dopamine agonists, use of antidepressants, and UPDRS III score. The GEE analysis was initially conducted using only a single covariate at a time (unadjusted model) and was subsequently performed using all covariates (adjusted model).

Significant longitudinal outcome changes in the NMSS scores were analyzed using the Friedman test. The strength of clinical progression of NMS was quantified with relative changes ([mean score _visit 2_−mean score _visit 1_]/mean score _visit 2_) and ES ([mean score _visit 1_−mean score _visit 2_]/SD _visit 1_) based on a previous report^38^. Values between 0.20 and 0.49, between 0.50 and 0.79, and ≥ 0.80 suggested that the ES was small, moderate, and large; respectively.

Forward binary logistic regression models were used to determine the association between the number of NMS at baseline and motor progression in PD. Faster motor progression observed during the follow-up was set as the dependent variable. The number of NMS at baseline was included as the independent variable. Multivariate analysis was used to adjust for the following variables: sex, age at enrollment, age of onset, disease duration, and UPDRS III score at baseline.

To exclude the effect of dopaminergic medication at baseline on the progression of NMS, we performed a sub-analysis of drug-naïve PD patients at baseline (*n* = 126).

### Reporting summary

Further information on research design is available in the Nature Research Reporting Summary linked to this article.

## Supplementary information

Supplementary Information

Reporting Summary

## Data Availability

Anonymized data can be obtained upon request from qualified investigators to replicate procedures and results.

## Code availability

No custom codes were used. All software or packages, their versions, relevant specification, and parameters are stated in the ‘Methods’ section.
