# Supplementary material for: Longitudinal evolution of non-motor symptoms in early Parkinson’s disease: a 3-year prospective cohort study
Source: NPJ Parkinsons Dis. 2021 Jul 15;7:58. doi: 10.1038/s41531-021-00207-5 (PMC8282608; doi:10.1038/s41531-021-00207-5)
Supplement: Supplementary file 1 — Supplementary Information [file 41531_2021_207_MOESM1_ESM.pdf]

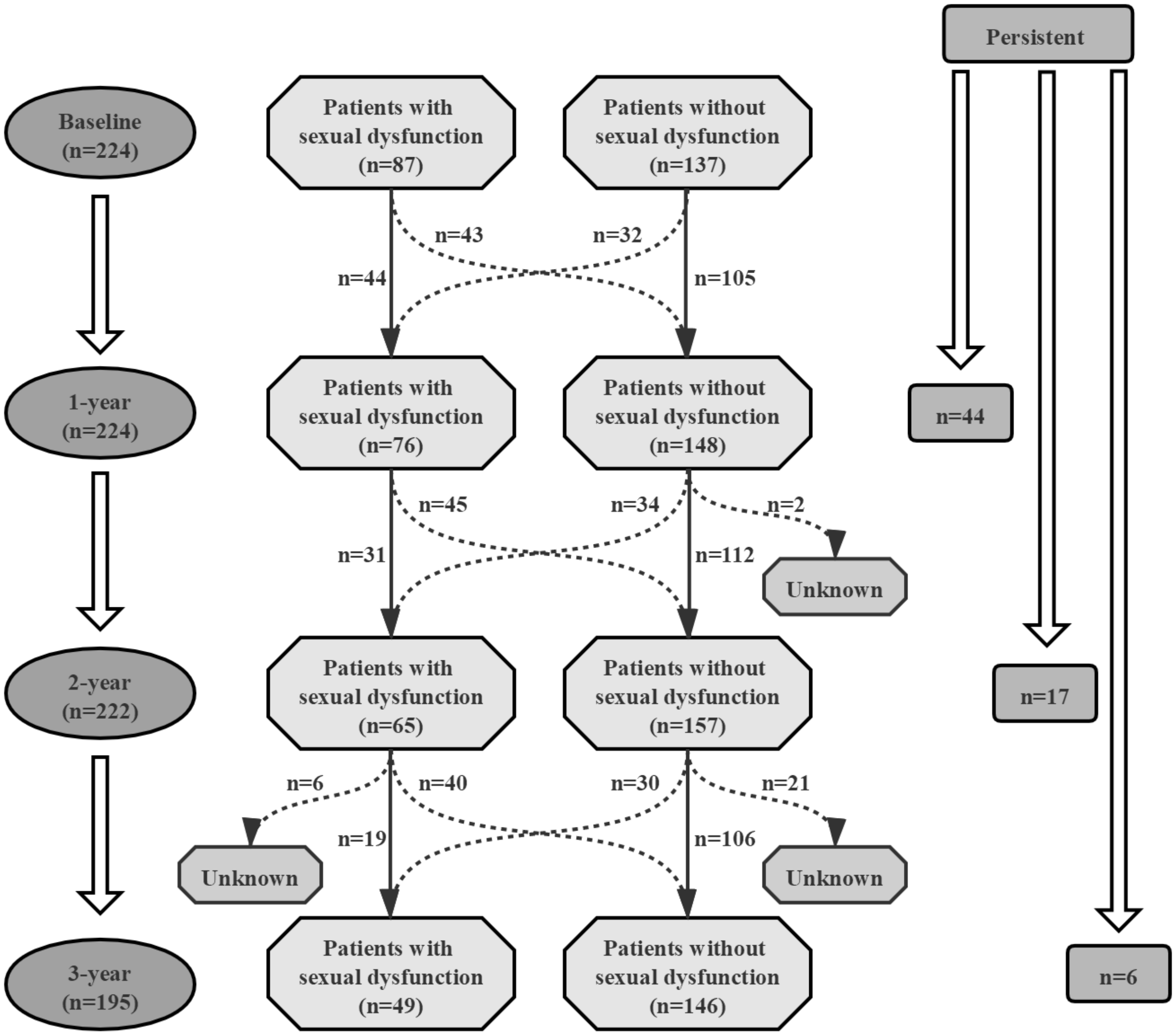
Persistent
Baseline
(n=224)
Patients with
sexual dysfunction
(n=87)
Patients without
sexual dysfunction
(n=137)
n=43
n=32
n=44
n=105
n=44
1-year
(n=224)
Patients with
sexual dysfunction
(n=76)
Patients without
sexual dysfunction
(n=148)
n=45
n=34
n=2
n=31
n=112
Unknown
n=17
2-year
(n=222)
Patients with
sexual dysfunction
(n=65)
Patients without
sexual dysfunction
(n=157)
n=6
n=40
n=30
n=21
n=19
n=106
Unknown
Unknown
3-year
(n=195)
Patients with
sexual dysfunction
(n=49)
Patients without
sexual dysfunction
(n=146)
n=6

Supplementary Figure 1 Longitudinal evolution of sexual dysfunction in patients with PD over 3 years

The numbers of sexual dysfunction in patients with PD were decreased from baseline to 3-year (87 to 49), and the occurrence of sexual dysfunction was not persistent. The numbers of persistent sexual dysfunction from baseline, 1-, 2-, and 3-year were 44, 17, and 6, respectively.

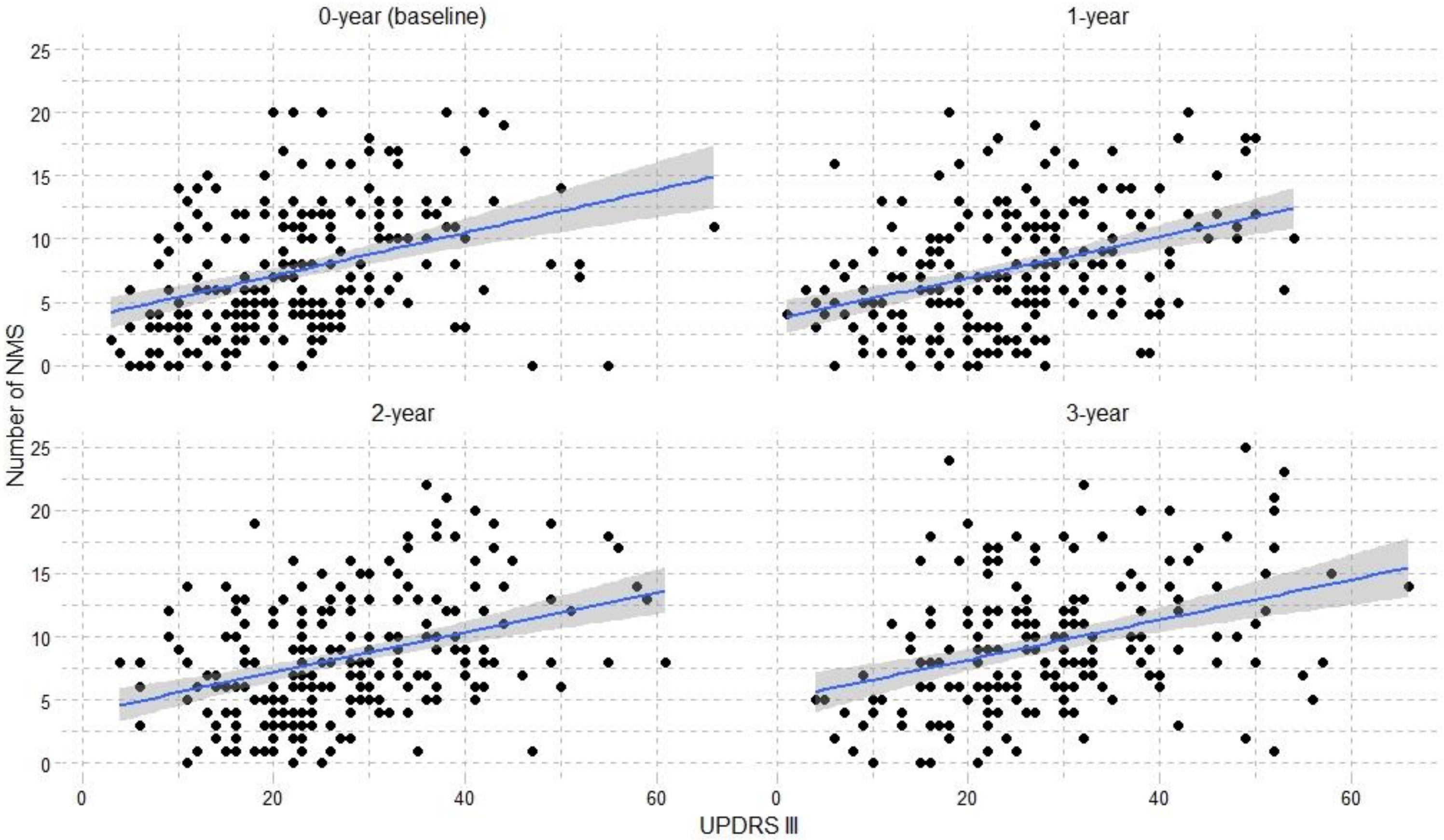

Supplementary Figure 2 Relationship between UPDRS III score and NMS number in patients with PD over 3 years

The fitted regression line indicated that the UPDRS III score increased with the number of NMS at each visit. Partial correlation with adjustment

for age: baseline (r = 0.336, $P < 0.001$); 1-year follow-up (r = 0.354, $P < 0.001$); 2-year follow-up (r = 0.359, $P < 0.001$); and 3-year follow-up (r = 0.330, $P < 0.001$).

Abbreviations: UDPRS, Unified Parkinson's Disease Rating Scale; NMS, non-motor symptoms; PD, Parkinson's disease.

Supplementary Table 1 Demographic and clinical features of PD patients

	Baseline	1-year	2-year	3-year
Number of samples	224	224	222	195
Age, years, mean (SD)	57.6 (11.1)	59.0 (11.1)	60.3 (11.1)	61.9 (11.4)
Disease duration, mean (SD)	1.5 (0.9)	2.9 (1.1)	4.2 (1.3)	5.3 (1.5)
Male sex, n (%)	121 (54.0)	121 (54.0)	119 (53.6)	108 (55.4)
Education, mean (SD)	11.1 (3.9)	11.1 (3.9)	11.1 (4.0)	11.1 (4.0)
LEDD, mg/day, mean (SD)	129.3 (175.5)	322.4 (164.2)	422.1 (201.0)	468.2 (255.8)
Use of levodopa, n (%)	79 (35.3)	156 (69.6)	178 (80.2)	158 (81.0%)
Use of dopamine receptor, n (%)	50 (22.3)	136 (60.7)	171 (70.0)	152 (77.9%)
Use of antidepressant, n (%)	10 (4.5)	9 (4.0)	18 (8.1)	18 (9.2)
FAB score, mean (SD)	16.7 (1.7)	16.6 (1.9)	16.5 (1.7)	16.4 (1.9)
MOCA score, mean (SD)	26.4 (2.5)	26.3 (3.0)	26.2 (2.9)	26.0 (3.1)
HAMD score, mean (SD)	8.3 (7.6)	7.4 (6.5)	7.8 (6.8)	8.0 (6.8)
HAMA score, mean (SD)	6.0 (5.8)	6.2 (5.9)	6.2 (5.3)	6.9 (6.0)
NMSS score, mean (SD)	28.0 (24.2)	25.7 (22.6)	27.2 (21.8)	31.9 (28.8)
UPDRS III score, mean (SD)	22.5 (10.8)	24.4 (10.9)	27.2 (11.2)	28.2 (11.7)

H&Y, median (quartiles)	2.0 (1.5, 2.0)	2.0 (2.0, 2.0)	2.0 (2.0, 2.0)	2.0 (2.0, 2.5)

Abbreviations: PD = Parkinson's disease; SD = standard deviation; LEDD = levodopa equivalent daily dose; FAB = Frontal Assessment Battery; MOCA = Montreal Cognitive Assessment; HAMD = Hamilton Depression Rating Scale; HAMA = Hamilton Anxiety Rating Scale; NMSS = Non-Motor Symptom Scale; UPDRS = Unified Parkinson's Disease Rating Scale.

Supplementary Table 2 Comparisons of baseline characteristics between patients with and without sexual dysfunction

	Baseline			1-year			2-year			3-year		
	With sexual dysfunction	Without sexual dysfunction	*P*-value	With sexual dysfunction	Without sexual dysfunction	*P*-value	With sexual dysfunction	Without sexual dysfunction	*P*-value	With sexual dysfunction	Without sexual dysfunction	*P*-value
Number of samples	87	137		76	148		65	157		49	146	
Age	59.0 (10.0)	56.7 (11.7)	0.142	60.9 (10.8)	58.0 (11.2)	0.068	59.0 (11.9)	60.8 (10.7)	0.269	64.0 (11.4)	61.2 (11.4)	0.135
Disease duration	1.5 (0.8)	1.5 (0.9)	0.632	2.8 (1.1)	3.0 (1.1)	0.333	4.2 (1.4)	4.2 (1.2)	0.940	5.3 (1.2)	5.4 (1.5)	0.889
Male sex	52 (60.0)	69 (50.4)	0.169	45 (59.2)	76 (51.4)	0.264	40 (61.5)	79 (50.3)	0.127	35 (71.4)	73 (50.0)	0.009*
Education	10.9 (3.9)	11.2 (4.0)	0.541	10.9 (3.9)	11.2 (4.0)	0.554	11.2 (3.7)	11.0 (4.0)	0.690	11.0 (4.1)	11.2 (4.0)	0.796
LEDD	137.6 (168.4)	124.0 (180.3)	0.573	325.7 (162.9)	320.7 (165.4)	0.830	404.2 (200.3)	429.5 (201.5)	0.395	519.6 (238.9)	450.9 (257.1)	0.101
Use of levodopa	34 (39.1)	45 (32.8)	0.341	59 (77.6)	97 (65.5)	0.062	53 (81.5)	125 (79.6)	0.744	45 (91.8)	113 (77.4)	0.026*
Use of dopamine agonist	21 (24.1)	29 (21.2)	0.603	39 (51.3)	97 (65.5)	0.039*	45 (69.2)	126 (80.3)	0.076	41 (83.7)	111 (76.0)	0.264
Use of antidepressant	4 (4.6)	6 (4.4)	1.000	3 (3.9)	6 (4.1)	1.000	3 (4.6)	15 (9.6)	0.220	4 (8.2)	14 (9.6)	1.000
FAB	16.7 (1.6)	16.7 (1.8)	0.726	16.3 (2.0)	16.7 (1.8)	0.128	16.5 (1.7)	16.5 (1.7)	0.898	16.0 (2.2)	16.6 (1.8)	0.069
MOCA	26.4 (2.5)	26.4 (2.5)	0.889	25.9 (3.0)	26.5 (3.0)	0.180	26.3 (2.8)	26.2 (2.9)	0.820	25.6 (3.4)	26.1 (3.0)	0.313
HAMD	9.9 (8.3)	7.3 (6.9)	0.016*	8.3 (6.9)	6.9 (6.3)	0.140	9.9 (7.5)	7.0 (6.2)	0.006*	10.9 (7.6)	7.0 (6.3)	0.002*

HAMA	7.1 (6.0)	5.2 (5.5)	0.021*	6.4 (5.8)	6.1 (5.9)	0.702	7.9 (6.0)	5.5 (4.9)	0.005*	8.8 (6.1)	6.2 (5.8)	0.010*
UPDRS III	23.5 (10.2)	21.9 (11.1)	0.267	26.3 (11.0)	23.5 (10.7)	0.060	28.1 (13.2)	26.9 (10.3)	0.519	33.6 (10.8)	26.5 (11.5)	<0.001*
H&Y stage	2.0 (2.0, 2.0)	2.0 (1.0, 2.0)	0.140	2.0 (2.0, 2.0)	2.0 (2.0, 2.0)	0.202	2.0 (2.0, 2.0)	2.0 (2.0, 2.0)	0.499	2.0 (2.0, 2.5)	2.0 (2.0, 2.0)	0.007*

Abbreviations: LEDD = levodopa equivalent daily dose; FAB = frontal assessment battery; MOCA = Montreal Cognitive Assessment; HAMD = Hamilton Depression Rating Scale; HAMA = Hamilton Anxiety Rating Scale; UPDRS = Unified Parkinson's Disease Rating Scale.

* Significant difference.

Supplementary Table 3 Number of patients with changed drugs use among different sexual dysfunction group

Sexual dysfunction	Levodopa				Dopamine agonist				Antidepressant			
	--	-+	+-	++	--	-+	+-	++	--	-+	+-	++
From baseline to 1-year (n=224)												
Group 1 (n=105)	30 (28.6)	41 (39.0)	5 (4.8)	29 (27.6)	26 (24.8)	53 (50.5)	10 (9.5)	16 (15.2)	99 (94.3)	1 (1.0)	2 (1.9)	3 (28.6)
Group 2 (n=32)	9 (28.1)	12 (37.5)	0	11 (34.4)	17 (53.1)	12 (37.5)	1 (3.1)	2 (6.3)	30 (93.8)	1 (3.1)	0	1 (3.1)
Group 3 (n=43)	14 (32.6)	12 (27.9)	2 (4.7)	15 (34.9)	13 (30.2)	21 (48.8)	2 (4.7)	7 (16.3)	39 (90.7)	1 (2.3)	2 (4.7)	1 (2.3)
Group 4 (n=44)	7 (15.9)	20 (45.5)	1 (2.3)	16 (36.4)	14 (31.8)	18 (40.9)	5 (11.4)	7 (15.9)	42 (95.5)	1 (2.3)	1 (2.3)	0
From 1-year to 2-year (n=222)												
Group 1 (n=112)	25 (22.3)	13 (11.6)	1 (0.9)	73 (65.2)	21 (18.8)	15 (13.4)	0	76 (67.9)	98 (87.5)	9 (8.0)	3 (2.7)	2 (1.8)
Group 2 (n=34)	6 (17.6)	7 (20.6)	0	21 (61.8)	6 (17.6)	8 (23.5)	4 (11.8)	16 (47.1)	32 (94.1)	1 (2.9)	0	1 (2.9)
Group 3 (n=45)	6 (13.3)	2 (4.4)	0	37 (82.2)	9 (20.0)	13 (28.9)	1 (2.3)	22 (48.9)	40 (88.9)	3 (6.7)	1 (2.3)	1 (2.3)
Group 4 (n=31)	6 (19.4)	3 (9.7)	0	22 (71.0)	7 (22.6)	8 (25.8)	3 (9.7)	13 (42.0)	29 (93.5)	1 (3.2)	1 (3.2)	0
From 2-year to 3-year (n=195)												
Group 1 (n=106)	14 (13.2)	7 (6.6)	12 (11.3)	73 (68.9)	12 (11.3)	8 (7.5)	17 (16.0)	69 (65.1)	94 (88.7)	2 (18.9)	2 (1.9)	8 (7.5)
Group 2 (n=30)	0	4 (13.3)	0	26 (86.7)	2 (6.7)	7 (23.3)	0	21 (70.0)	24 (80.0)	1 (3.3)	2 (6.7)	3 (10.0)
Group 3 (n=40)	7 (17.5)	0	0	33 (82.5)	6 (15.0)	5 (12.5)	0	29 (72.5)	35 (87.5)	2 (5.0)	1 (2.5)	2 (5.0)
Group 4 (n=19)	4 (21.1)	0	0	15 (78.9)	5 (26.3)	2 (10.5)	1 (5.3)	11 (57.9)	19 (100.0)	0	0	0

Group 1: patients had no sexual dysfunction at both two visits.

Group 2: patients had no sexual dysfunction at visit 1 but had sexual dysfunction at visit 2.

Group 3: patients had sexual dysfunction at visit 1 but had no sexual dysfunction at visit 2.

Group 4: patients had sexual dysfunction at both two visits.

--: patients did not use drugs at both two visits.

-+: patients used drugs at visit 1 but did not at visit 2.

+-: patients did not use drugs at visit 1 but used at visit 2.

++: patients used drugs at both two visits.

Supplementary Table 4 Factors associated with the presence of sexual dysfunction in patients with PD

	Unadjusted model			Adjusted model		
	OR	95%CI	*P*-value	OR	95%CI	*P*-value
Age	1.009	0.994-1.025	0.245	1.011	0.996-1.027	0.160
Disease duration	0.888	0.813-0.969	0.008*	0.860	0.777-0.951	0.003*
Male sex	1.605	1.147-2.246	0.006*	1.646	1.162-2.331	0.005*
Education	0.990	0.947-1.035	0.654			
LEDD	1.000	0.999-1.000	0.325			
Use of levodopa	1.216	0.900-1.642	0.203			
Use of dopamine agonist	0.691	0.510-0.935	0.017*	0.816	0.580-1.149	0.244
Use of antidepressant	0.710	0.392-1.288	0.260			
FAB	0.939	0.859-1.026	0.164			
MOCA	0.975	0.920-1.034	0.397			
HAMD	1.054	1.031-1.078	<0.001*	1.059	1.019-1.100	0.003*
HAMA	1.049	1.021-1.078	<0.001*	0.995	0.951-1.040	0.823
UPDRS III	1.019	1.004-1.033	0.013*	1.012	0.995-1.028	0.165

Abbreviations: PD = Parkinson's disease; LEDD = levodopa equivalent daily dose; FAB = frontal assessment battery; MOCA = Montreal Cognitive Assessment;

HAMD = Hamilton Depression Rating Scale; HAMA = Hamilton Anxiety Rating Scale; UPDRS = Unified Parkinson’s Disease Rating Scale.

* Significant difference.
